# Case report—CARMAT: the first experience with the Aeson bioprosthetic total artificial heart as a bridge to transplantation in a case of post-infarction ventricular septal rupture

**DOI:** 10.3389/fcvm.2023.1211365

**Published:** 2023-09-29

**Authors:** Katharina Huenges, Bernd Panholzer, Jochen Cremer, Assad Haneya

**Affiliations:** Department of Cardiovascular Surgery, UKSH, Kiel, Germany

**Keywords:** total artificial heart, VSD, myocardial infarction, heart transplantation, heart failure

## Abstract

**Background:**

Post-infarction ventricular septal defects remain one of the most feared complications after myocardial infarction with high mortality rates. In special cases, surgical or interventional treatment strategies are technically not feasible and do not always lead to a good outcome.

**Case presentation:**

A 58-year-old male patient in cardiogenic shock with a very large ventricular septal (VSD) defect (4.9 cm × 5 cm) due to myocardial infarction was presented in our department. Acute stabilization was achieved using peripheral venoarterial extracorporeal membrane oxygenation (VA-ECMO) support. Neither surgical nor interventional therapy was considered as a sufficient option due to the unsuitable anatomy of the VSD and the patient was listed for heart transplantation. After 2 weeks on ECMO, bleeding and infectious complications occurred. Due to organ shortage, urgent implantation of the bioprosthetic total artificial heart (TAH) Aeson device (CARMAT) remained the only useful strategy to achieve a mid- or long-term bridge to transplantation. After successful implantation and good recovery with the Aeson device, the patient was transplanted 4 weeks after implantation.

**Conclusion:**

Post-infarction ventricular septal defects are highly challenging and are commonly associated with a poor prognosis. The implantation of the new Aeson TAH device is a promising therapeutic option, allowing a safe and long-term bridging to heart transplantation.

## Background

The diagnostic and therapeutic options for myocardial infarction have improved in the past decades. But despite all, in extended myocardial infarction, one of the most dreaded complications is the development of ventricular septum defects. While the occurrence is rare, it is one of the most severe impairing clinical conditions for a patient (1, 2). The cause of the ventricular septum defect is, e.g., transmural myocardial tissue necrosis leading to fragile myocardial tissue and subsequent rupture, causing a left-to-right blood shunt. Depending on the shunt volume, hemodynamics can deteriorate up to life-threatening cardiogenic shock. For many years, the primary surgical septum repair using felt-pledged sutures or pericardial patch reconstructions was the only available treatment option in those patients. For selected cases, interventional approaches by closing the VSD with occlude devices have been performed in the past as well. All in all, the prognosis of patients with post-infarction VSD remains poor.

In recent years, the concept of stabilizing those patients was primarily either with peripheral or central mechanical circulatory support (MCS) devices, i.e., VA-ECMO therapies. Secondary to repair, the VSD which is a surgical strategy, or in selected cases, an interventional septal occlusion device after a certain myocardial recovery time was introduced and adopted by many centers (3, 4). With the MCS, hemodynamic stabilization can be achieved in most of the patients, gaining time for consolidation of myocardial tissue and myocardial recovery and secondary organ recovery after cardiogenic shock but not in all patients. Sufficient and persistent hemodynamic stabilization can be accomplished with MCS strategies. Furthermore, in some patients, pulmonary-venous backflow or pulmonary flooding may lead to pulmonary edema, facilitating respiratory failure and pneumonia. Additionally, the optimal length of MCS support is difficult: The MCS devices can only be used as a bridge to therapy during a certain time frame before MCS-associated side effects, above all bleeding and infectious complications, may occur. Not infrequently, as physicians, we are faced with the indissoluble dilemma that the patient would require more time for myocardial recovery (for tissue regeneration and scarring process) to increase the probability of a stable VSD correction, but time on MCS is already too long to prevent patients from life-threatening device-related complications. Hence, short-term MCS such as VA-ECMO is not expedient. Furthermore, some post-infarction VSDs are anatomically unsuitable for both—surgical or interventional—closures, i.e., in very large defects or with a close vicinity or even direct involvement of the atrioventricular valve area. In those patients, heart transplantation remains the only useful therapeutic option. Due to organ shortage, the time on the waiting list for heart transplantation is many weeks, months, or even more likely years in certain countries.

Unfortunately, there is a lack of promising results in using durable MCS devices such as ventricular assist devices (VAD) (either left or right VAD or biventricular VAD) in post-infarction VSD patients. On the one hand, those systems do not address the ventricular defect, and on the other hand, their certain cardiac anatomy is not suitable for durable VAD implantation and function. Another rescue procedure, the implantation of extracorporeal biventricular support systems, faces many potential complications and has severe implications for the quality of life of the patients, especially regarding the long-term aspects since mostly hospitalization in ICU is required (5).

In 2021, the bioprosthetic Aeson TAH (CARMAT SA, Vélizy, France) was introduced commercially and implanted successfully in several patients with biventricular heart failure as a bridge to heart transplant.

The first implantation of the Aeson CARMAT TAH in a patient with severe post-infarction VSD is presented in this case report.

## Case description

A 58-year-old male patient with no known comorbidities was admitted to the chest pain unit of our hospital in cardiogenic shock with an acute ST-segment elevation myocardial infarction (STEMI) and a deteriorating hemodynamic condition. A coronary angiography performed upon admission revealed severe 3-vessel coronary artery disease with occlusion of the dominant right coronary artery (RCA) and severe stenosis of the left anterior descending artery (LAD) and the circumflex branch (RCX), and revascularization with implantation of 3 drug-eluting-stents (DES) in the RCA was performed. During left ventricular (LV) angiography, a large ventricular septum defect to the atrioventricular valve plane was noticed, causing a massive shunt volume. Echocardiography confirmed the large defect with right ventricular distention and severely reduced biventricular function. Despite adequate catecholamines and i.v. inotropic support, it was not possible to achieve sufficient stabilization of the patient in severe cardiogenic shock, so peripheral transfemoral V-A ECMO implantation was unavoidable. Immediately after establishing the V-A ECMO, the hemodynamics stabilized and the overall clinical condition slightly improved. With non-invasive ventilation therapy, intubation was avoidable, and under full V-A ECMO support, the impaired secondary organ functions (the patient suffered initially from acute liver and renal failure) were normalized again.

**Figure 1:**
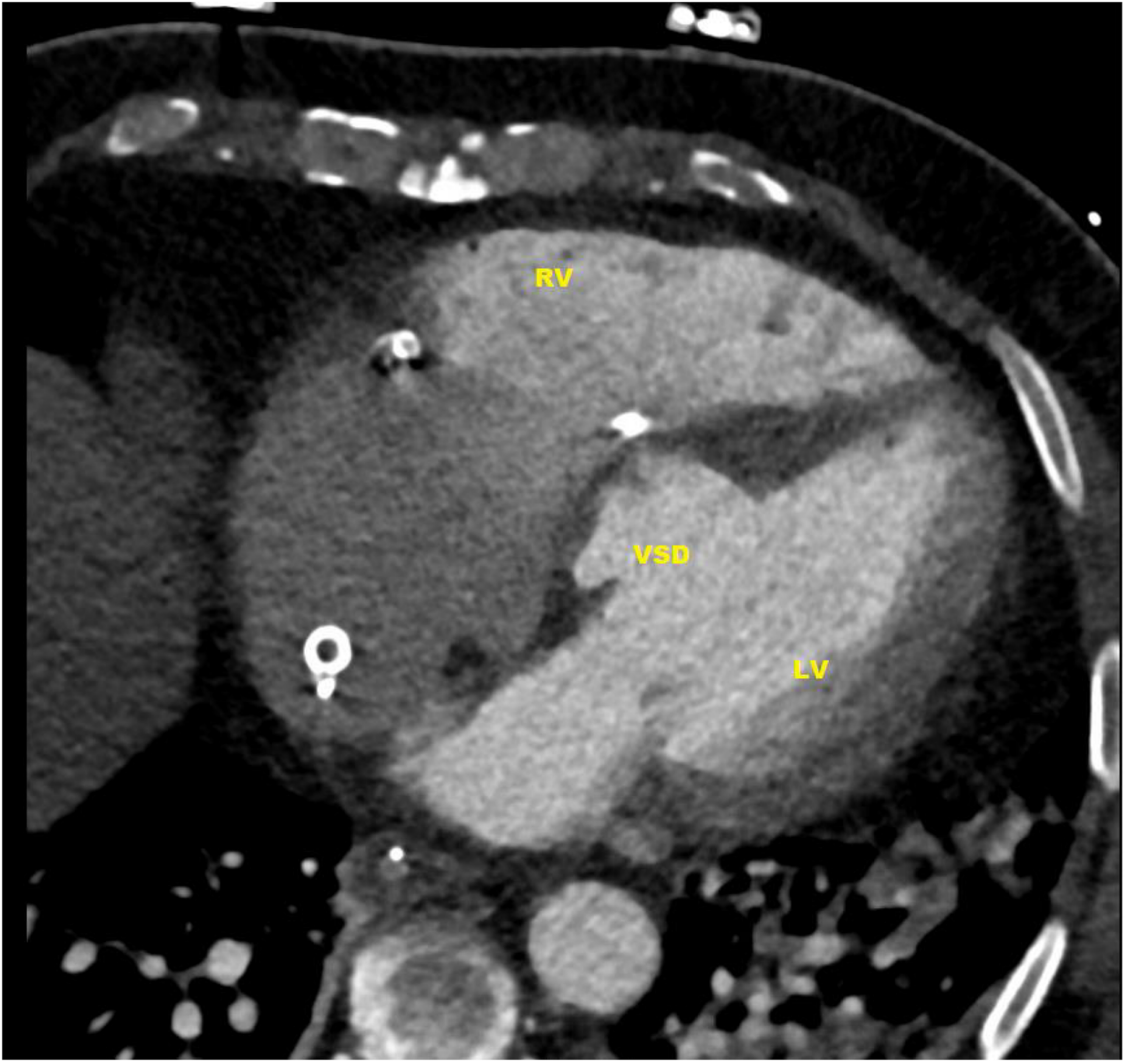
CT-scan: ventricular septal defect (VSD) directly under the valvular plane, volume-loaded left and right ventricle (LV, RV).

**Figure 2:**
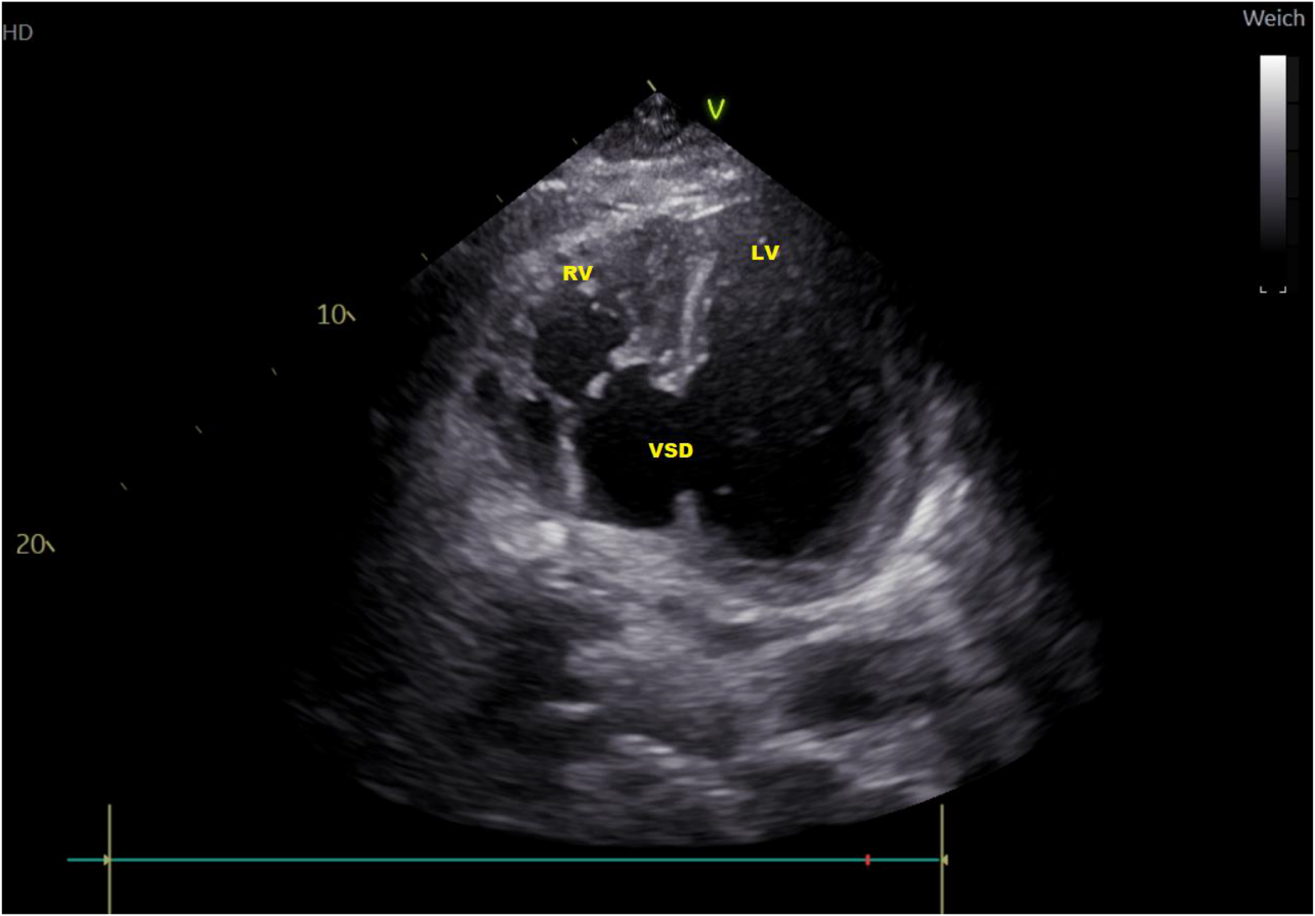
Transthoracic echocardiography atypical two-chamber view: ventricular septal defect (VSD), loaded left and right ventricle (LV, RV).

In subsequent echocardiography and cardiac computed tomography, the full dimensions of the ventricular septum defect were visualized. We discussed the VSD extensively with our adult and pediatric cardiologist colleagues and our congenital cardiac surgeons. Since dimensions were measured with 4.9 × 5 cm and its very close localization to the atrioventricular valve area (Figures 1, 2) not leaving a sufficient rim for placing either sutures or patch-sutures, the VSD was considered untreatable. In light of very few prospects for success, we considered a surgical VSD exploration and attempt of closure as way too risky for the patient.

Since no contraindications were found and the patient was fully awake and vigilant, we discussed the option of heart transplantation as the only reasonable therapy with him and his family. After consent and according to the decision of our interdisciplinary transplant conference team, the patient was listed at Eurotransplant (ET, Leiden, Netherlands) on high-urgency status.

After 12 days of ECMO therapy, we were faced with the fact that there was no suitable donor organ on the horizon and that the remaining number of complication-free days for our patient was fading. Furthermore, ECMO-related bleeding and infectious complications occurred. Hence, the patient was urgently screened for potential Aeson TAH implantation. A computed tomography scan of the chest confirmed anatomic suitability and the implant procedure was scheduled.

**Figure 3:**
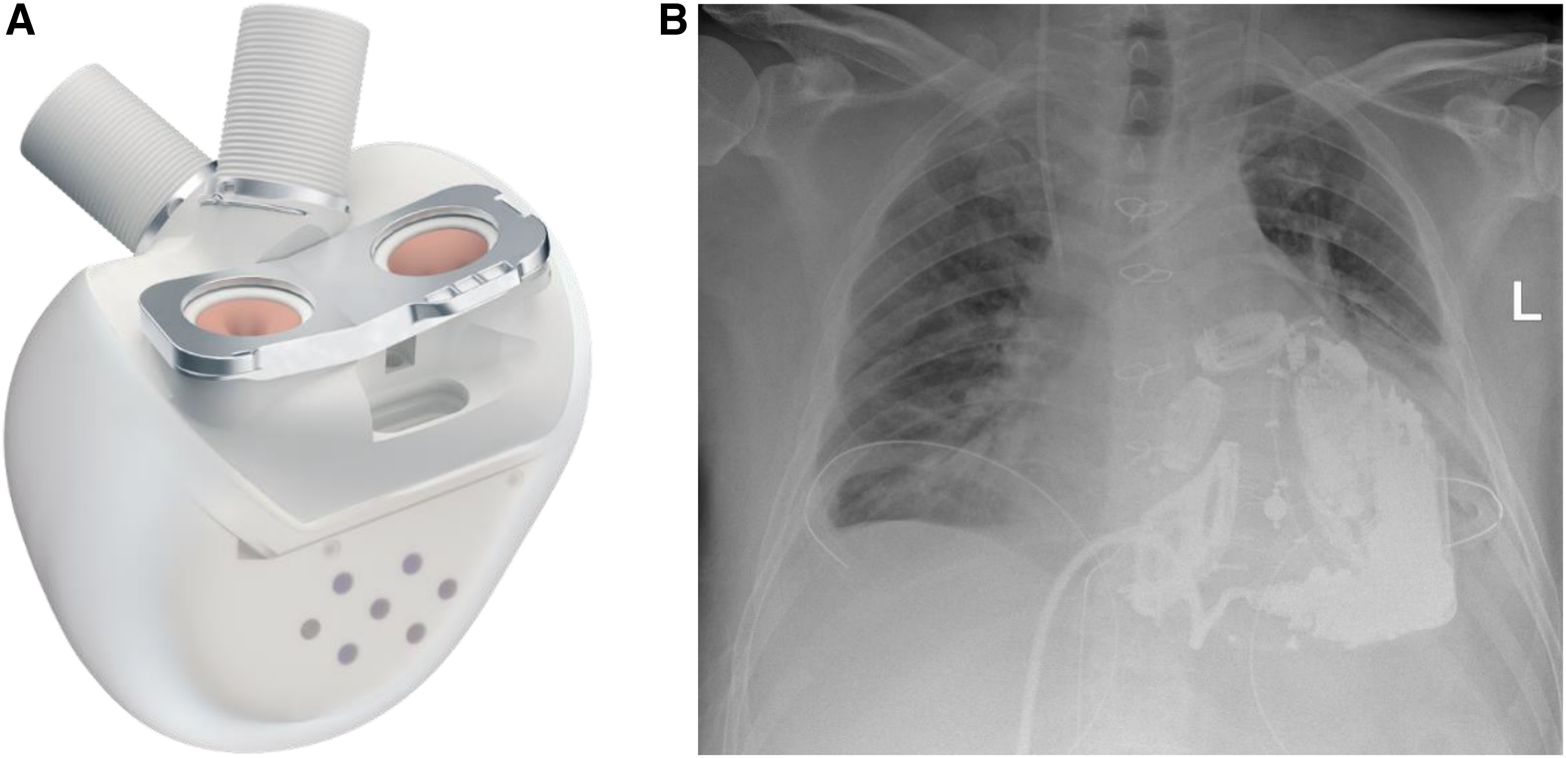
(**A**) The Aeson TAH device, courtesy of CARMAT (SA, Vélizy, France). (**B**) Chest x-ray of the patient after Aeson TAH implantation.

The surgical procedure started with a surgical preparation of the groin vessels followed by median sternotomy and aortic and bicaval cannulation. Cardiopulmonary Bypass (CPB) with central cannulation was initiated and the ECMO cannulas were removed surgically. At full CPB flow, the aorta was cross-clamped, as distally as possible. The native ventricles were excised one centimeter above the atrio-ventricular plane. To reduce the risk of air embolism, CO_2_ was flushed into the pericardial space. Two bioprosthetic atrial cuffs with central openings were sewn to the annuli with two layers of running sutures. A rectangular titanium interface was placed and secured to the cuffs. The Aeson device, mounted with four biological valves and two outflow conduits, was attached to the atrial interface (Figure 3). The Dacron outflow conduits were then connected to the pulmonary and the aorta with running sutures and re-enforcing patches. The driveline was tunneled through the right rectus muscle with skin exit at the lower right abdominal quadrant. The device was then connected to the portable controller. The TAH was first passively filled and de-aired with a vent placed in the ascending aorta. Pump beat rate and stroke volume were gradually increased while CPB support was weaned. Shortly after careful volume administration, the TAH was able to achieve stable cardiac output, and a substantial decrease in the catecholamines was achieved.

**Figure 4:**
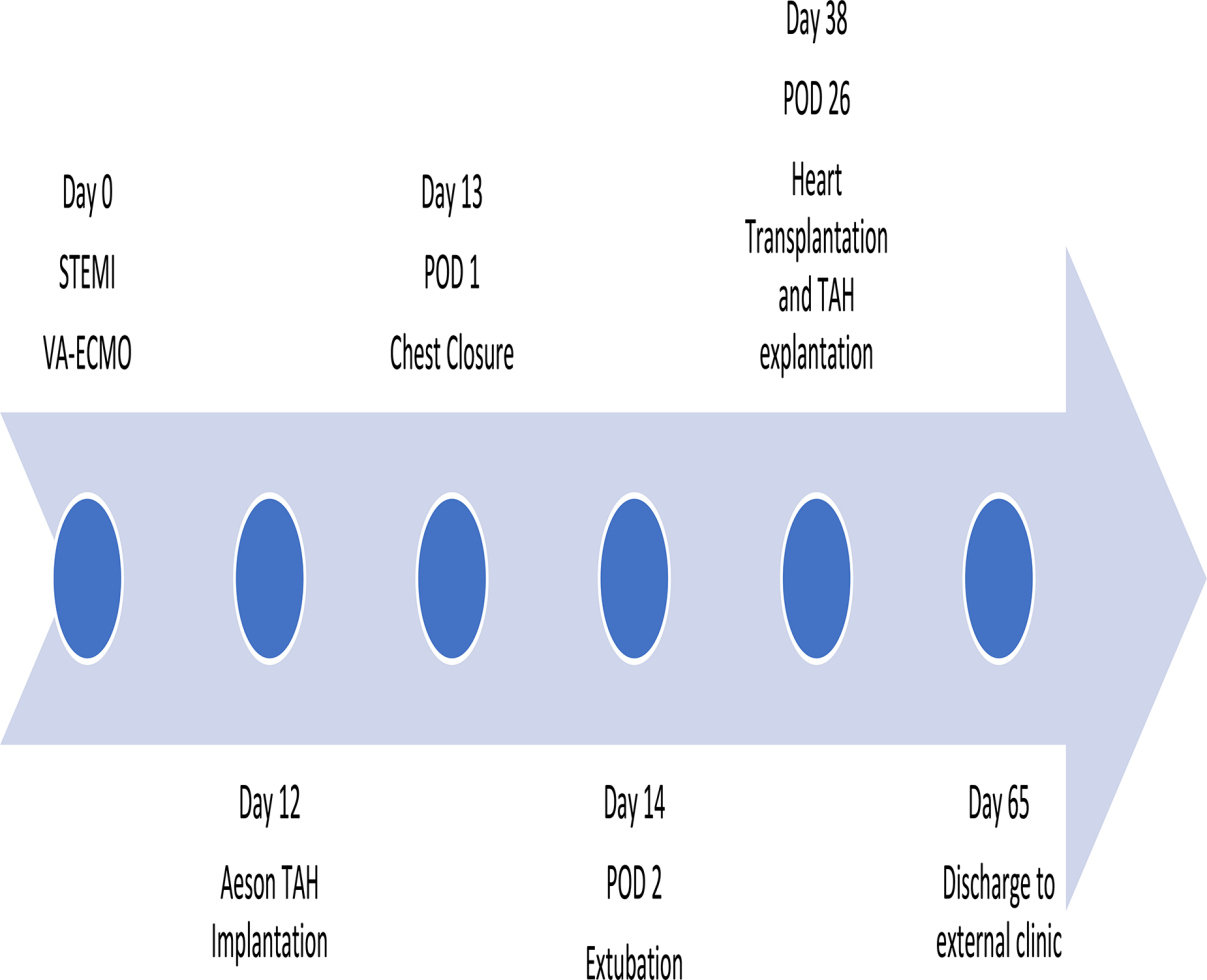
Timeline.

To avoid tamponade and its hemodynamic consequences, definite chest closure was postponed to 1 day after the surgery (Figure 4). There were no relevant bleeding complications. To assist in the recovery of pre-existing hepatic and renal dysfunction related to cardiogenic shock, a combined liver-renal dialysis therapy (ADVOS, ADVITOS GmbH, Munich, Germany) was installed during the first day post-implant.

Due to its hemocompatibility blood-contacting surfaces and minimized shear stress (6), the Aeson TAH device only requires a mono thrombocyte-inhibitor application (i.e., 100 mg acetylic salicylic acid daily) and low-dose thrombosis prophylaxis (i.e., tinzaparin s.c.).

**Figure 5:**
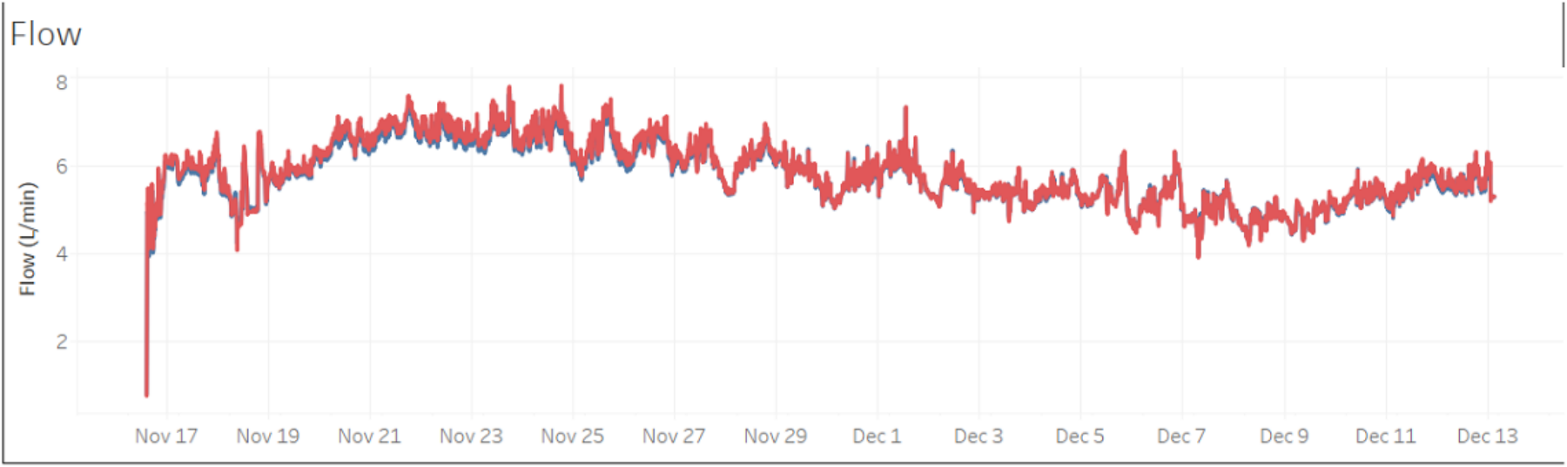
Flow pattern (flow in L/min) of the first 26 days with the TAH support in the VSD patient.

The TAH was functioning in its standard automatic mode, with flow adjustments to over 5 L/min based on changes in preload detected by pressure sensors inside the device (Figure 5). The patient was extubated 2 days after the implantation. Mobilization was possible from day 3 and the patient was soon able to walk with support on ward-level. Extensive respiratory therapy was required to treat atelectasis of the left lower lung lobe caused by the size of the device.

After sufficient general recovery, the patient was reconsidered for heart transplantation. Additionally, atelectasis of the lower lobe of the left lung recurred with impeding complex pneumonia. Retroactively, the Aeson diameters seemed to have a higher impact on the left lung ventilation as initially measured in the screening CT scan. Around 4 weeks (26 days) after the TAH implantation, a suitable donor organ was accepted for the patient. During the heart transplantation procedure, it was remarkably easy to explant the Aeson TAH device due to its smooth surface preventing adhesions. Orthotopic bicaval heart transplantation was performed, and after removal of all patches and vascular prosthesis of the Aeson device, the situs was normal to any primary heart transplantation situs, and normal direct anastomosis of the left atrium, superior and inferior caval veins, pulmonary artery and ascending aorta was performed. The patient was transferred to the ICU in a stable condition with moderate catecholamine support. Sedation could be stopped on the first postoperative day, and the patient could be extubated immediately afterward.

Despite his initial very good course, in the following days, the patient developed an imminent respiratory failure due to left-sided pneumonia requiring re-intubation. For his respiratory recovery, a tracheotomy was necessary and the patient was discharged to a weaning rehabilitation center 27 days after heart transplantation. The decannulation was possible 1 week after he was transferred, and additionally, his renal function was fully recovered, and no renal replacement therapy was necessary. At the 6-month post-transplant follow-up visit in our transplant center, we were able to see a healthy patient in good general condition, with a stable heart function and the full ability to lead a self-independent daily life including his normal professional everyday life.

## Conclusion

The possibility of treating patients suffering from severe post-infarction ventricular septum defects with long-term total artificial heart support may serve as a safe strategy and a good therapeutic option in these high-risk patients. This new TAH therapy with low-intensity anticoagulation and automated flow regulation enables stabilization and improvement of patients after severe myocardial infarction, facilitating a safe heart transplantation procedure.

## Data Availability

The original contributions presented in the study are included in the article/Supplementary Material, further inquiries can be directed to the corresponding author.

## References

[1] Treille de Grandsaigne H Bouisset F Porterie J Biendel C Marcheix B Lairez O Incidence, management, and prognosis of post-ischaemic ventricular septal defect: insights from a 12-year tertiary centre experience. Front Cardiovasc Med. (2022) 9:1066308. 10.3389/fcvm.2022.1066308 36561773 PMC9763320

[2] Cinq-Mars A Voisine P Dagenais F Charbonneau É Jacques F Kalavrouziotis D Risk factors of mortality after surgical correction of ventricular septal defect following myocardial infarction: retrospective analysis and review of the literature. Int J Cardiol. (2016) 206:27–36. 10.1016/j.ijcard.2015.12.011 26773765

[3] Trivedi KR Aldebert P Riberi A Mancini J Levy G Macia JC Sequential management of post-myocardial infarction ventricular septal defects. Arch Cardiovasc Dis. (2015) 108(5):321–30. 10.1016/j.acvd.2015.01.005 25754906

[4] Giblett JP Matetic A Jenkins D Ng CY Venuraju S MacCarthy T Post-infarction ventricular septal defect: percutaneous or surgical management in the UK national registry. Eur Heart J. (2022) 43(48):5020–32. 10.1093/eurheartj/ehac511 36124729

[5] Gregoric ID. Total artificial heart in patients with post-infarction ventricular septal defect. Ann Cardiothorac Surg. (2020) 9(2):116. 10.21037/acs.2020.01.04 32309161 PMC7160633

[6] Smadja DM Ivak P Pya Y Latremouille C Gustafsson F Roussel JC Intermediate-dose prophylactic anticoagulation with low molecular weight heparin is safe after bioprosthetic artificial heart implantation. J Heart Lung Transplant. (2022) 41(9):1214–7. 10.1016/j.healun.2022.05.017 35715318

